# Comparison of Social Needs Among US Insured Adults Before and During the Early Phase of the COVID-19 Pandemic

**DOI:** 10.1001/jamanetworkopen.2021.46700

**Published:** 2022-02-25

**Authors:** Douglas W. Roblin, Syed I. Khalid, Christopher Rouillard

**Affiliations:** 1 Mid-Atlantic Permanente Research Institute, Kaiser Permanente, Rockville, Maryland; 2 Department of Neurosurgery, University of Illinois at Chicago; 3 The Carle Illinois College of Medicine, University of Illinois, Urbana-Champaign

## Abstract

This cross-sectional study uses survey data linked with electronic health record data to compare the prevalence of social needs among US insured adults before vs during the early phase of the COVID-19 pandemic.

## Introduction

Onset of the COVID-19 pandemic was associated with changes in need for social assistance among patients of integrated delivery systems in the US.^1^ Few studies have investigated the association of the pandemic with patients’ social needs overall or by need category.^2,3^ In this cross-sectional study, the prevalence of social needs during the early pandemic period (January to June 2020) was compared with the prevalence in a matched sample in a prepandemic period (January to June 2019) from Kaiser Permanente Mid-Atlantic States (KPMAS) health care system.

## Methods

The KPMAS health care system provides comprehensive medical services to approximately 780 000 residents of Washington DC; Baltimore, Maryland; and the surrounding Maryland and Virginia areas. The Your Current Life Situation (YCLS) survey^4^ was administered in-person and online from March 2016 through June 2020 to a convenience sample of patients who physicians and case managers believed had social needs that should be addressed for optimizing patient care. The YCLS survey records of respondents aged 18 or older were linked with electronic health record data. The institutional review board of KPMAS reviewed, approved, and monitored the study protocol. Because the YCLS survey was administered during routine care, the institutional review board waived the requirement of informed consent. This study followed the Strengthening the Reporting of Observational Studies in Epidemiology (STROBE) reporting guideline.

Responses to the YCLS survey (eMethods and eTables 1 and 2 in the Supplement) were classified as need for assistance with housing or utilities payments, transportation, food payment or meal preparation, and medical services or medication payments. Covariates for propensity score matching were measured from electronic health record data and included respondent age and insurance plans at time of survey administration, gender, race and ethnicity (patient self-reported), Charlson Comorbidity Index^5^ in the 365 days before survey administration, and socioeconomic status represented by the national percentile of the area disadvantage index^6^ of the respondent’s residential Census tract in the survey year. A propensity score model matched prepandemic respondents to early pandemic respondents based on these covariates. We used χ^2^ statistics to compare the prevalence of social needs before vs during the pandemic and during the first 3 months (January to March 2020) vs second 3 months (April to June 2020) of the pandemic. Significance was set at *P* ≤ .05 for 2-sided tests. Analyses were conducted using SAS, version 9.4 (SAS Institute).

## Results

Before matching, the sample consisted of 18 544 respondents (10 849 [58.5%] female; mean [SD] age, 60.7 [18.5] years) (after removing 2055 surveys owing to repeated surveys for the same respondent and/or missing relevant survey items or covariates). The matched sample included 4373 respondents each in the prepandemic period (2523 [57.7%] female; mean [SD] age, 58.9 [19.0] years) and early pandemic period (2510 [57.4%] female; mean [SD] age, 58.7 [18.9] years) (Table 1). Respondents were well matched on covariates after matching.

**Table 1.** Sample Distributions Before and During the COVID-19 Pandemic Before and After Propensity Score Matching

Characteristic	Before matching			After matching		
	Respondents, No. (%)		*P* value	Respondents, No. (%)		*P* value
	Before the pandemic (n = 14 169)	During the pandemic (n = 4375)		Before the pandemic (n = 4373)	During the pandemic (n = 4373)	
Age, y						
≥75	3599 (25.4)	984 (22.5)	<.001	990 (22.6)	984 (22.5)	.97
65-74	3281 (23.2)	862 (19.7)		850 (19.4)	862 (19.7)	
50-64	3989 (28.2)	1273 (29.1)		1266 (29.0)	1273 (29.1)	
18-49	3300 (23.3)	1256 (28.7)		1267 (29.0)	1254 (28.7)	
Gender						
Female	8339 (58.9)	2510 (57.4)	.08	2523 (57.7)	2510 (57.4)	.78
Male	5830 (41.1)	1865 (42.6)		1850 (42.3)	1863 (42.6)	
Race and ethnicity						
Hispanic	1093 (7.7)	376 (8.6)	<.001	352 (8.1)	376 (8.6)	.81
Non-Hispanic						
Black	7125 (50.3)	2067 (47.3)		2067 (47.3)	2067 (47.3)	
White	4446 (31.4)	1279 (29.2)		1289 (29.5)	1279 (29.3)	
Other or unknown^a^	1505 (10.6)	653 (14.9)		665 (15.2)	651 (14.9)	
Charlson Comorbidity Index						
≥1 Class	6839 (48.3)	1750 (40.0)	<.001	1762 (40.3)	1750 (40.0)	.79
None	7330 (51.7)	2625 (60.0)		2611 (59.7)	2623 (60.0)	
Health plan						
Medicaid	2309 (16.3)	932 (21.3)	<.001	933 (21.3)	930 (21.3)	.67
Medicare	6295 (44.4)	1806 (41.3)		1785 (40.8)	1806 (41.3)	
High deductible	1168 (8.2)	391 (8.9)		400 (9.2)	391 (8.9)	
Standard HMO	4210 (29.7)	1187 (27.1)		1210 (27.7)	1187 (27.1)	
Other	187 (1.3)	59 (1.4)		45 (1.0)	59 (1.4)	
Area disadvantage index, quartile						
Most disadvantaged	4262 (30.1)	1424 (32.6)	<.001	1422 (32.5)	1422 (32.5)	.99
Lower middle	2604 (18.4)	804 (18.4)		814 (18.6)	804 (18.4)	
Upper middle	4009 (28.3)	1178 (26.9)		1171 (26.8)	1178 (26.9)	
Most advantaged	3254 (23.3)	969 (22.2)		966 (22.1)	969 (22.2)	

Abbreviation: HMO, health maintenance organization.

^a^ Other racial and ethnic groups included American Indian, Alaska Native, Asian, Native Hawaiian, Other Pacific Islander, other, and declined to state.

Between the prepandemic and early pandemic periods, the prevalence of transportation assistance need decreased (834 [19.1%] vs 679 [15.5%]; *P <* .001) (Table 2). The prevalence of housing or utilities payment, food payment or meal preparation, and medical services or medication payment assistance needs remained unchanged.

**Table 2.** Respondents in the Propensity Score Matched Data Set Expressing Social Needs Before and During the COVID-19 Pandemic

Period	Housing or utilities payment assistance		Transportation assistance		Food payment or meal preparation assistance		Medical service or medication payment assistance	
	Respondents, No. (%)^a^	*P* value	Respondents, No. (%)^a^	*P* value	Respondents, No. (%)^a^	*P* value	Respondents, No. (%)^a^	*P* value
**Before vs during the pandemic**								
Before the pandemic (n = 4373)	515 (11.8)	.57	834 (19.1)	<.001	779 (17.8)	.96	775 (17.7)	.59
During the pandemic (n = 4373)	498 (11.4)		679 (15.5)		781 (17.9)		756 (17.3)	
**First vs second 3 mo of the pandemic**								
January to March 2020 (n = 2496)	248 (9.9)	<.001	438 (17.6)	<.001	405 (16.2)	<.001	430 (17.2)	.90
April to June 2020 (n = 1877)	250 (13.3)		241 (12.8)		376 (20.0)		326 (17.4)	

^a^ Percentages calculated across rows.

Between January to March 2020 and April to June 2020, the prevalence of transportation assistance need decreased (438 [17.6%] vs 241 [12.8%]; *P* < .001) (Table 2). During this period, the prevalence of housing or utilities payment (248 [9.9%] vs 250 [13.3%]; *P* < .001) and food payment or meal preparation (405 [16.2%] vs 376 [20.0%]; *P* < .001) assistance needs increased. The prevalence of medical services or medication payment assistance needs remained unchanged.

## Discussion

In this cross-sectional study, between the prepandemic and early COVID-19 pandemic periods, the prevalence of housing or utilities payment assistance, food payment or meal preparation, and medical service or medication payment assistance needs remained unchanged among respondents to the YCLS survey. The prevalence of transportation assistance need decreased. From the first 3 months of the pandemic to the second 3 months of the pandemic, the prevalence of housing or utilities payment and food payment or meal preparation assistance needs increased.

KPMAS patient assistance programs, instituted before the pandemic, have offered assistance in arranging or subsidizing transportation to clinics, waiving medication co-payments, and scheduling home delivery of prepared meals during the pandemic. In addition, KPMAS shifted appointment availability from in-person to virtual care during the pandemic. Whether these factors were associated with changes in the prevalence of needs was beyond the scope of our study.

Whether loss of employment or reduced working hours during the pandemic were associated with decreased household income and increased need for housing or utilities payment assistance was also beyond the scope of our study. Government or nongovernmental organizations, not health care systems, typically focus on addressing such social needs.

A limitation of our study is that estimates of the prevalence of social needs before and during the pandemic were based on self-reports from a convenience sample of insured adults in an integrated delivery system with relevant patient assistance programs. Findings might not be generalizable to other health care systems, community settings, or social needs (eg, assistance with dependent care).

The role of health care systems in addressing social needs remains understudied. Our study may provide a framework for assessing the association of the COVID-19 pandemic with social assistance needs in other insured populations.

## Notes

Supplement.
eMethods.
**eTable 1.** YCLS Items Indicating Social Needs Relevant to This Study **eTable 2.** YCLS Item Responses Indicating a Specific Social Need
