# Supplementary material for: Comparison of Social Needs Among US Insured Adults Before and During the Early Phase of the COVID-19 Pandemic
Source: JAMA Netw Open. 2022 Feb 25;5(2):e2146700. doi: 10.1001/jamanetworkopen.2021.46700 (PMC8881764; doi:10.1001/jamanetworkopen.2021.46700)
Supplement: Supplement. — eMethods. eTable 1. YCLS Items Indicating Social Needs Relevant to This Study eTable 2. YCLS Item Responses Indicating a Specific Social Need [file jamanetwopen-e2146700-s001.pdf]

Supplemental Online Content

Roblin DW, Khalid SI, Rouillard C. Comparison of social needs among US insured adults before and during the early phase of the COVID-19 pandemic. *JAMA Netw Open.* 2022;5(2):e2146700. doi:10.1001/jamanetworkopen.2021.46700

eMethods.

eTable 1. YCLS Items Indicating Social Needs Relevant to This Study

eTable 2. YCLS Item Responses Indicating a Specific Social Need

This supplemental material has been provided by the authors to give readers additional information about their work.

© 2022 Roblin DW et al. *JAMA Network Open.*

eMethods.

Derivation of Social Needs Measures Used in This Study from the Your Current Life Situation Survey Items and Responses.

Step 1. The study team first reviewed all items from the Your Current Life Situation (YCLS) survey and selected the subset of items which seemed to be relevant to measurement of social needs relevant to our study (eTable 1).

Step. 2. We reviewed the possible responses to each YCLS item (eTable 1) and selected responses which seemed to indicate those responses specific to each social need. Some items had responses indicative of only on social need; other items had responses indicative of several social needs. eTable 2 displays the YCLS item and response that the study team determined was indicative of a social need. For example, we determined that social need due to "housing costs or residential stability" was indicated by the following YCLS items and responses:

- Q1 "5: Temporarily staying with a relative or friend" or "6: Temporarily staying in a shelter or homeless", OR
- Q2 "1: Condition of housing", or "2: Feeling safe", or "3: Ability to pay for housing or utilities", or "4: Lack of more permanent housing", OR
- Q3 "2: Housing", OR
- Q8 "2: Housing"

© 2022 Roblin DW et al. *JAMA Network Open.*

eTable 1. YCLS Items Indicating Social Needs Relevant to This Study.

YCLS Item Number	YCLS Item as Presented in the YCLS	Social Need Indicated by YCLS Item
Q1	"Which of the following best describes your current living situation?"	Housing Costs / Residential Stability
Q2	"Do you have any concerns about your current living situation, like housing conditions, safety, and costs? (Y/N and if Y specify)"	Housing Costs / Residential Stability
Q3	"In the past 3 months, did you have trouble paying for the following?"	Housing Costs / Residential Stability<br>Utility Costs<br>Transportation Costs / Availability<br>Food Costs / Availability<br>Medical Services Costs
Q4	"In the past 3 months, how often have you worried that your food would run out before you had money to buy more?"	Food Costs / Availability
Q5	"Has lack of transportation kept you from medical appointments or doing things for daily living?"	Transportation Costs / Availability
Q8	"Which of the following would you like to receive help with at this time?"	Housing Costs / Residential Stability<br>Utility Costs<br>Transportation Costs / Availability<br>Food Costs / Availability<br>Medical Services Costs

© 2022 Roblin DW et al. *JAMA Network Open.*

eTable 2. YCLS Item Responses Indicating a Specific Social Need.

YCLS Item	YCLS Item Responses Indicating a Specific Social Need				
	Housing Costs/Residential Stability	Utility Costs	Transportation Costs / Availability	Food Costs / Availability	Medical Services Costs
Q1: Describe your current living situation	"5: Temporarily staying with a relative or friend"<br>"6: Temporarily staying in a shelter or homeless"	N/A	N/A	N/A	N/A
Q2: Concerns about current living situation	"1: Condition of housing"<br>"2: Feeling safe"<br>"3: Ability to pay for housing or utilities"<br>"4: Lack of more permanent housing"	"3: Ability to pay for housing or utilities"	N/A	N/A	N/A
Q3: Trouble paying	"2: Housing"	"3: Utility bills (electric, etc.)"	"6: Transportation"	"1: Food"	"5: Medical needs (medicines, doctor, etc.)"
Q4: Worried about food	N/A	N/A	N/A	"2: Sometimes"<br>"3: Often"<br>"4: Very often"	N/A
Q5: Lack of transportation	N/A	N/A	"1: Kept me from medical appointments or from getting medications"<br>"2: Kept me from doing things needed for daily living"	N/A	N/A
Q8: Would like to receive help	"2: Housing"	"4: Utilities (heat, electricity, water, etc.)"	"3: Transportation"	"1: Food"	"5: Medical care, medicine, medical supplies"<br>"6: Dental services"<br>"7: Vision services"

© 2022 Roblin DW et al. *JAMA Network Open.*

© 2022 Roblin DW et al. *JAMA Network Open.*
